# Supplementary material for: A data mining paradigm for identifying key factors in biological processes using gene expression data
Source: Sci Rep. 2018 Jun 13;8:9083. doi: 10.1038/s41598-018-27258-8 (PMC5998123; doi:10.1038/s41598-018-27258-8)
Supplement: Supplementary file 1 — Supplemental materials [file 41598_2018_27258_MOESM1_ESM.docx]

Peng Yu, PhD

Department of Electrical and Computer Engineering & TEES-AgriLife Center for Bioinformatics and Genomic Systems Engineering,

Texas A&M University,

College Station, TX 77843, USA

Tel: 1-979-845-7441

Fax: 1-979-845-6259

Email: [pengyu.bio@gmail.com](mailto:lzheng.chn@gmail.com)

**A data mining paradigm for identifying key factors in biological processes using gene expression data**

Jin Li^1,2^, Le Zheng^1^, Akihiko Uchiyama^3^, Lianghua Bin^4^, Theodora M. Mauro^5^, Peter M. Elias^5^, Tadeusz Pawelczyk^6^, Monika Sakowicz-Burkiewicz^6^, Magdalena Trzeciak^7^, Donald Y. M. Leung^4^, Maria I. Morasso^3^ and Peng Yu^1,2,*^

^1^Department of Electrical and Computer Engineering & ^2^TEES-AgriLife Center for Bioinformatics and Genomic Systems Engineering, Texas A&M University, College Station, TX 77843, USA

^3^Laboratory of Skin Biology, National Institute for Arthritis and Musculoskeletal and Skin Diseases, National Institutes of Health, Bethesda, MD, USA

^4^Department of Pediatrics, National Jewish Health, Denver, Colorado, USA

^5^Dermatology Service, Veterans Affairs Medical Center, and Department of Dermatology, UCSF, San Francisco, California, USA

^6^Department of Molecular Medicine, Medical University of Gdansk, Gdansk, Poland

^7^Department of Dermatology, Venerology and Allergology, Medical University of Gdansk, Gdansk, Poland

*Correspondence: [pengyu.bio@gmail.com](mailto:lzheng.chn@gmail.com)

**SUPPLEMENTAL INFORMATION**

**Text S1. Comparisons on the curated datasets with respect to epidermal development**

To assess the ability of the paradigm, differentially expressed genes using individual comparisons were compared to top identified genes. For individual comparisons, the genes were ordered by contrasts for the increased process or by negative contrasts for the decreased process. Because there were 295 genes in the epidermis development GO term (accession GO:0008544), the same number of genes was selected as top genes in each individual comparison. Further, 295 top genes were selected for the combined comparisons. Some genes tied at the rank of 295, so 24 instances of random sampling (to generate the same number of observations as individual comparisons) were performed within these tied genes to keep only a total of 295 genes. Within each of these sets of 295 genes, the number of the genes in the epidermis development GO term was recorded. To test the difference of epidermal development genes between individual comparisons and top identified genes, one-sided Wilcoxon test was applied over the recorded number of epidermal development genes.

**Text S2. Phylogenetics-based GO analysis**

Because the function of *SBSN* has not been elucidated, it is important to derive an unbiased indication regarding its biological function. For this purpose, a GO analysis based on a gene set derived by a phylogenetic approach was performed using the following procedure. Co-evolved genes of human *SBSN* were first detected using the human-centric binary phylogenetic matrix from Clustering by Inferred Models of Evolution (CLIME) ^9^. The human-centric phylogenetic matrix in CLIME was built by searching the protein sequence of each gene in humans against the protein sequences in the rest of 138 fully sequenced eukaryotic organisms ^10^ and in a “prokaryote” outgroup of 502 prokaryotic species using BLASTP ^11^. In this matrix, rows are human genes, and columns are the 138 eukaryotic organisms together with the “prokaryote” outgroup. Each element in the matrix is binary, which takes 1 if the human protein sequence of the gene in the row is similar to the sequence of a protein in the species of the column; otherwise it takes 0. Then, the Fisher’s exact test was applied to evaluate the significance that each gene was co-evolved with *SBSN* among 138 eukaryotic organisms and the “prokaryote” outgroup. A total of 59 genes were co-evolved with *SBSN* under $p<1.0\times{10}^{-6}$. These 59 co-evolved genes were used to screen for the enriched GO terms using Fisher’s exact test (with the null hypothesis *H*_0_: log-odds-ratio < 2) with the genes appearing in all human GO terms as background ^12^.

The GO analysis resulted in three significantly enriched GO terms related to epidermal development: keratin filament, intermediate filament, and intermediate filament cytoskeleton (**Figure S4**). For example, keratin filament has shown to be critical in the formation of skin disorders ^13^. These enriched GO terms identified by the co-evolved genes of *SBSN* indicate a potentially critical role of *SBSN* in epidermal development.

**Text S3. Expression increase of *SBSN* upon epidermal differentiation**

To evaluate the gene expression changes of *SBSN* upon epidermal differentiation, a microarray dataset (GSE52651) measured in a 7-day time-course keratinocyte differentiation experiment was analyzed. Human progenitor keratinocytes were seeded onto devitalized dermis to enable keratinocyte differentiation into fully stratified epithelium, which captured dynamic changes in tissue regeneration ^14^. With log2 transformation and quantile normalization of raw probe expression values, **Figure S5** shows increased expression of *SBSN* upon epidermal differentiation starting from day 1. The early increase of its expression values upon the induction of differentiation indicates a potentially critical role of *SBSN* in epidermal differentiation.

**Text S4. Expression of *SBSN* transcripts in AD skin**

The expression of the full-length transcript of *SBSN* (v1) was significantly decreased in AD lesional skin compared to nonlesional skin and healthy controls (**Figure 4c**). The transcript v2 (NM_198538.3) of *SBSN* showed significantly decreased levels in AD lesional skin compared to nonlesional skin, but not controls. However, the transcript v3 (NM_001166035.1) showed no significant expression changes in AD lesional skin compared to nonlesional skin and controls, even though nonlesional skin showed an increased expression compared to controls (**Figure S7**). The v2 and v3 *SBSN* transcript variants had lower expression compared to the full-length transcript (v1) ($\sim10\%$ and $<1\%$ of v1 in healthy controls). Because the v2 and v3 *SBSN* transcript variants were much less abundant compared to the full-length transcript (v1), the full-length transcript of *SBSN* may be the *SBSN* isoform critical in AD.

**Text S5. Clustering analysis using the affinity distance metric based on Fisher’s exact test**

To investigate the relationship of the 24 experimental comparisons in the curated datasets, clustering analysis was performed using an affinity distance metric. The affinity distance metric was derived from an affinity score matrix calculated in the paradigm (**Figure 1**). An affinity score (annotated as $+1/-1/0$ or NA) of a gene in an experimental comparison examines the relatedness of the gene to a biological process. To evaluate the similarity of the results of two experimental comparisons, a $3\times3$ contingency table, labeled as $+1/0/-1$, was tabulated by counting the number of genes from the two columns in the affinity score matrix; the table then was collapsed into two $2\times2$ tables such that the enrichment of the genes having $+1$s or $-1$s in both experimental comparisons could be tested using Fisher’s exact test. The geometric mean of the two *p-*values calculated from the two $2\times2$ tables corresponding to $+1$s and $-1$s was considered the affinity distance between the two experimental comparisons. A smaller affinity distance indicates a closer relationship between the two experimental comparisons. To examine the relationships among the 24 experimental comparisons in our curated datasets, the affinity distances were calculated for all pairs of 24 comparisons and were saved in an affinity distance matrix. Then, hierarchical clustering with complete linkage was applied to this matrix.

**Text S6. Empirical distribution of consensus score**

To determine the cutoff of consensus scores, simulations were performed to generate the empirical distribution. Specifically, the consensus scores for all genes and 24 comparisons in epidermal development were used to construct an original score matrix, with rows as genes and columns as comparisons. To perform the simulation, the affinity scores for each comparison (each column) were permutated. After all columns were permutated, the consensus scores were calculated for each row. A total of 10,000 iterations of simulation were executed to generate the empirical distribution of consensus scores.

**Text S7. DEG analysis using microarray data**

For each of the curated human microarray datasets in **Table S1**, DEG analysis was performed as described below. To map microarray probes to gene symbols, the probe sequences were aligned to the transcript sequences of the GENCODE human annotation (release 25) ^1^ using Bowtie (version 1.1.2) ^2^ with an exact match. The probes aligned to multiple genes were discarded. The raw microarray probe data were then rank-normalized by transforming the raw probe values to ranks scaled to [0, 1] by dividing the total number of probes in each platform. These scaled ranks were transformed by the variance-stabilizing transformation (VST) ^3^. The resulted VST values were fit using linear models with adapted FDR in contrasts ^4,5^. The DEGs were identified as FDR $\leq0.05$.

**Text S8. DEG analysis using RNA-Seq data**

For DEG analysis using RNA-Seq data, raw full-length of the single-end or the first end of the paired-end reads were first aligned to the transcriptome sequences annotated in GENCODE (mouse release M12) ^6^ eliminating pseudogenes using STAR (version 2.5.3a) ^7^ ignoring multiple alignment reads. A count table was tabulated of the number of reads aligned to each gene, discarding those reads aligned to multiple genes. Genes with low counts were filtered out from the count table. Normalization and DEG were conducted using DESeq2 ^8^. FDR-adjusted *q*-values were computed using the Benjamini-Hochberg procedure ^4^. The DEGs were identified as |log2-fold-change| $>0.5$ and $q<0.05$.

**Figure S1. Heatmap of the top genes (consensus score** $\boldsymbol{\geq6}$**) in epidermal development derived from 24 experimental comparisons of the curated datasets.**

To identify the candidate genes that are potentially important in epidermal development, the paradigm was applied to the curated datasets. A total of 81 top genes (consensus score $\geq6$) revealed a set of candidate genes involved in epidermal development. Each column in the heatmap represents one of the 24 experimental comparisons in the curated datasets. For example, “ZNF750/-; GSE32685; PHK” represents the dataset (GSE32685) in which *ZNF750* was knocked down in primary human keratinocytes. Each row corresponds to a gene that was examined in those experimental comparisons. The colors yellow/blue/black/white correspond to the affinity scores $+1/-1/0/$NA, respectively. These 81 top genes showed an affinity in epidermal tissues, demonstrating potential roles of these genes in epidermal development.

**Figure S2. The majority of identified genes were not annotated in the epidermis development GO term.**

To evaluate the effectiveness of the paradigm in identifying new factors in epidermal development, the top identified genes were overlapped with the genes in the epidermis development GO term (GO:0008544). These identified genes were extracted using consensus score thresholds from $\geq6$ to $\geq10$. The green and red bars depict the number of total identified genes given the threshold and the number of the genes in the epidermis development GO term, respectively. The majority of identified genes (consensus score $\geq6$) were not in the epidermis development GO term.

**Figure S3. The paradigm revealed an increased number of epidermal development genes.**

To demonstrate the power of the paradigm, differentially expressed genes derived from individual comparisons were compared to the top ranked genes using all the comparisons. One-sided Wilcoxon test was used to test the significance of the difference between the number of epidermal development genes from two approaches. (***: *p*-value $<0.001$)

**Figure S4. GO terms enriched in the co-evolved genes of *SBSN*.**

Three GO terms, keratin filament, intermediate filament, and intermediate filament cytoskeleton, were significantly enriched in the 59 co-evolved genes of *SBSN* in humans.

**Figure S5. Expression changes of *SBSN* in human keratinocytes upon epidermal differentiation.**

To investigate the gene expression changes of *SBSN* upon epidermal differentiation, a time-course microarray dataset was used to measure the expression values of *SBSN*. Human keratinocytes were treated to induce differentiation for discrete time points of seven days. The boxplot shows the normalized log2-expression values of the 11 probes in the microarray mapped to *SBSN* with two biological replicates measured per day. The expression of *SBSN* was significantly up-regulated in days 1 to 7 compared with day 0 (*t*-test using linear contrast *p*-value $<2.2\times{10}^{-16}$).

**Figure S6. Enriched GO terms of identified genes in CIT.**

The identified genes (consensus score $\geq6$) were used to screen GO terms, and the figure depicts the enriched GO terms. The *p-*values and odds ratio from Fisher’s exact test were recorded for each GO term.

**Figure S7. Expression values of *SBSN* transcript v2 and v3 in AD skins.**

To evaluate the expression changes of *SBSN* in AD skins, expression values were measured in AD skins for the *SBSN* transcripts via RT-PCR. The expression levels were normalized by the expression levels of *G6PD*. (a) As shown in Figure 4c, the *SBSN* transcript v1 showed significantly decreased expression levels in AD lesional skins compared to AD nonlesional and control skins. (b) The *SBSN* transcript v2 showed significantly different expression changes between AD lesional versus nonlesional skins and nonlesional versus control skins. (c) The *SBSN* transcript v3 showed significantly different expression changes between AD nonlesional versus control skins (***: *p*-value $<0.001$, **: *p-*value $<0.01$, *: *p*-value $<0.05$).

**Figure S8. Dendrogram of the experimental comparisons in the curated datasets.** To identify the relationship among the 24 experimental comparisons of our curated datasets, a hierarchical clustering analysis was performed using the affinity distance metric. The experimental comparisons were annotated by perturbed genes, types of perturbation, tissue and cell types, and ArrayExpress/GEO accession IDs with citations. The experimental comparisons were also colored by their tissue and cell types—cancer cells (red), epidermal cells (gold), epithelial cells (green), organotypic tissue (blue), and trabecular meshwork cells (purple).


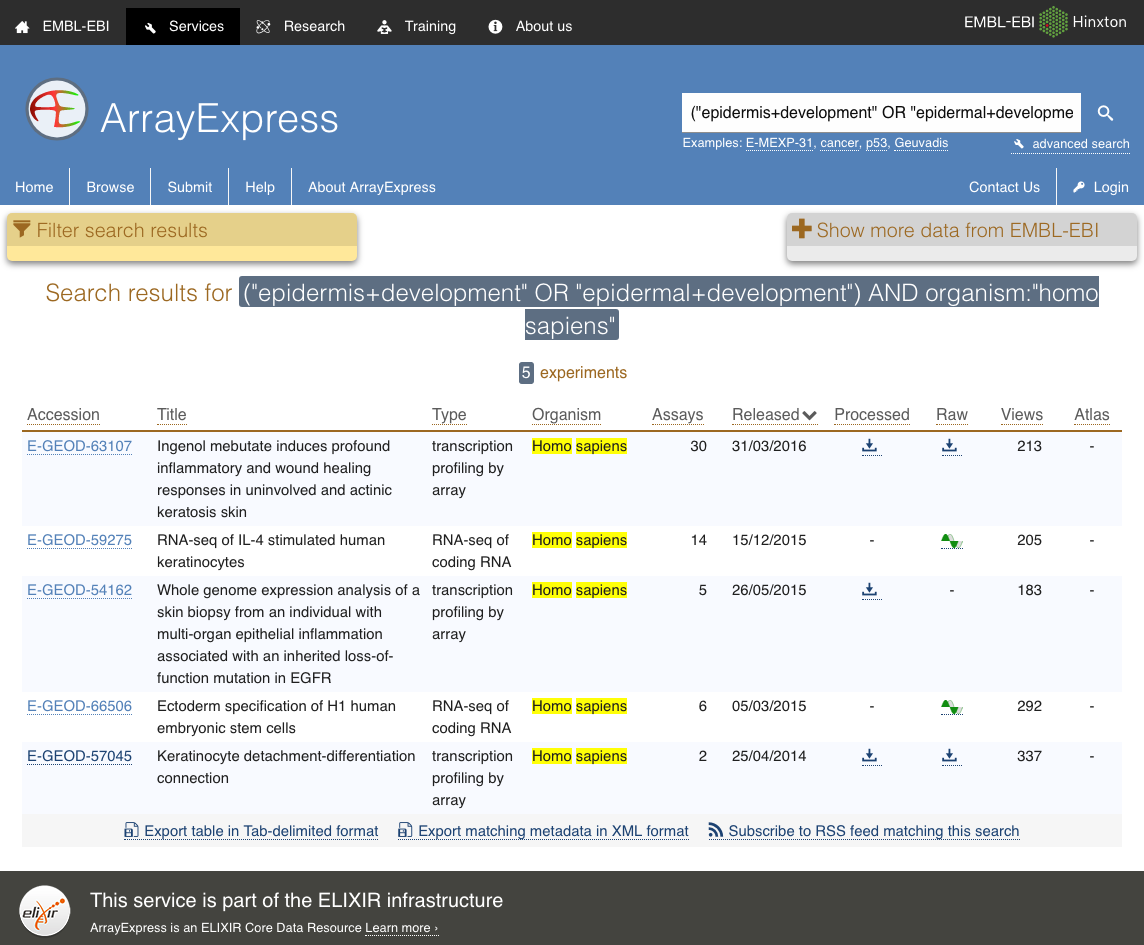


**Figure S9. Screenshot of datasets retrieved from ArrayExpress for epidermal development.**

To collect gene expression data for epidermal development, ArrayExpress was searched using the text regarding epidermal development. The screenshot depicts five retrieved datasets for human gene expression data.

**Figure S10. Empirical distribution of consensus score in simulated epidermal development and CIT.**

The figure shows the empirical probability mass function (pmf) of consensus scores. Among 10,000 iterations of simulation, observed consensus scores ranged from –6 to 6 for both epidermal development and CIT. The normalized frequency is shown for the distribution of consensus scores.

**Figure S11. Number of genes for each consensus score in epidermal development and CIT.**

Among the 24 comparisons for epidermal development and CIT, consensus scores were calculated for all measured genes. The bars represent the number of genes for corresponding consensus scores.

| **Table S1. Result of dataset curation on GEO by the epidermis development GO term genes** | | | | | |
| --- | --- | --- | --- | --- | --- |
| **GSE No.** | **Perturbed Gene** | **Perturbation** | **Experiment Tissue** | **Abbreviation** | **Tissue Type** |
| GSE37637 | EXOSC9 | Overexpressed | Primary human keratinocytes | PHK | Epidermal tissue |
| GSE71017 | GRHL2 | Knockdown | Ovarian cancer cell line OVCA429 | OVCA429 | Cancer cell |
| GSE37049 | GRHL3 | Knockdown | Primary human normal neonatal keratinocytes | NHEK | Epidermal tissue |
| GSE32685 | KLF4 | Knockdown | Primary neonatal keratinocytes | HEKn | Epidermal tissue |
|  | ZNF750 |  |  |  |  |
| GSE1676 | RELA | Knockdown | HEK 293 | HEK 293 | Organotypic tissue |
| GSE62454 | RUNX1 | Knockdown | LNCaP cell line | LNCaP | Cancer cell |
| GSE24778 | RUNX1 | Knockdown | K562 cells | K562 | Cancer cell |
| GSE8640 | TFAP2A | Knockdown | MCF7 | MCF7 | Cancer cell |
|  | TFAP2C |  |  |  |  |
| GSE28448 | SMAD4 | Knockdown | HMEC-TR | HMEC-TR | Epithelial cell |
|  | TIF1 |  |  |  |  |
| GSE33495 | TP63 | Knockdown | Primary neonatal keratinocytes | HEKn | Epidermal tissue |
|  | TP63 |  |  |  |  |
|  | TP63 |  |  |  |  |
| GSE38039 | ZNF750 | Knockdown | HaCaT cells | HaCaT | Cancer cell |
| E-MTAB-1833 | CUX1 | Knockdown | Loucy cells | Loucy | Organotypic tissue |
| GSE27275 | PITX2 | Knockdown | Trabecular meshwork (TM) tissue from eye | TM | Trabecular meshwork cell |
| E-MTAB-900 | RELA | Knockdown | HEK293T | HEK293T | Organotypic tissue |
| GSE70940 | SMAD4 | Overexpressed (8hr) | Pancreatic ductal adenocarcinoma (PDAC) cell line BxPC3 | PDAC | Cancer cell |
|  |  | Overexpressed (24hr) |  |  |  |
|  |  | Overexpressed (48hr) |  |  |  |
| GSE28558 | SNAI1 | Knockdown | A549 | A549 | Cancer cell |
| GSE44203 | TFAP2C | Knockdown | MCF7 | MCF7 | Cancer cell |

**Table S4. Eight enriched cornified envelope genes in *Sbsn* knockdown mouse differentiating keratinocyte cultures.**

| Cnfn |
| --- |
| Lce1g |
| Lce1h |
| Lce3c |
| Lce3d |
| Lce3e |
| Sprr2d |
| Sprr2e |

**Table S5. Ten gene expression datasets of adipose tissue upon cold exposure.**

| **GEO Accession** | **Experimental Tissue** | **Platform** |
| --- | --- | --- |
| GSE13432 | White adipose tissue | Affymetrix Mouse Genome 430 2.0 Array |
| GSE40486 | Brown adipose tissue;  Skeletal muscle | Illumina mouseRef-8 v1.1 expression beadchip |
| GSE44138 | Brown adipose tissue;  White adipose tissue;  Liver | Illumina Mouse Ref-6 V1 |
| GSE51080 | Brown adipose tissue;  Mesenteric white adipose tissue;  Posterior subcutaneous white adipose tissue | Affymetrix Mouse Genome 430 2.0 Array |
| GSE63031 | Interscapular brown adipose tissue;  Inguinal white adipose tissue;  Epididymal white adipose tissue | Illumina Genome Analyzer II |
| GSE64909 | Brown adipose tissue | Illumina MouseWG-6 v2.0 R2 expression beadchip |
| GSE70437 | Interscapular brown adipose tissue | Illumina HiSeq 1500 |
| GSE74062 | Epididymal white adipose tissue | Affymetrix Mouse Exon 1.0 ST Array |
| GSE74899 | Inguinal white adipose tissue | Affymetrix Mouse Transcriptome Array 1.0 |
| GSE77534 | Brown adipose tissue | Illumina HiSeq 2000 |

**References:**

1 Harrow, J. *et al.* GENCODE: the reference human genome annotation for The ENCODE Project. *Genome Res* **22**, 1760-1774 (2012).

2 Langmead, B., Trapnell, C., Pop, M. & Salzberg, S. L. Ultrafast and memory-efficient alignment of short DNA sequences to the human genome. *Genome biology* **10**, R25 (2009).

3 Durbin, B. P., Hardin, J. S., Hawkins, D. M. & Rocke, D. M. A variance-stabilizing transformation for gene-expression microarray data. *Bioinformatics* **18**, S105-S110 (2002).

4 Benjamini, Y. & Hochberg, Y. Controlling the False Discovery Rate: A Practical and Powerful Approach to Multiple Testing. *Journal of the Royal Statistical Society. Series B (Methodological)* **57**, 289-300 (1995).

5 ZHENG, L. & YU, P. *brt: Biological Relevance Testing, <*[*https://cran.r-project.org/web/packages/brt/index.html*](https://cran.r-project.org/web/packages/brt/index.html)*>*, 2017).

6 Harrow, J. *et al.* GENCODE: producing a reference annotation for ENCODE. *Genome Biol* **7 Suppl 1**, S4 1-9, doi:10.1186/gb-2006-7-s1-s4 (2006).

7 Dobin, A. *et al.* STAR: ultrafast universal RNA-seq aligner. *Bioinformatics* **29**, 15-21, doi:10.1093/bioinformatics/bts635 (2013).

8 Love, M. I., Huber, W. & Anders, S. Moderated estimation of fold change and dispersion for RNA-seq data with DESeq2. *Genome Biol* **15**, 550, doi:10.1186/s13059-014-0550-8 (2014).

9 Li, Y., Calvo, S. E., Gutman, R., Liu, J. S. & Mootha, V. K. Expansion of biological pathways based on evolutionary inference. *Cell* **158**, 213-225, doi:10.1016/j.cell.2014.05.034 (2014).

10 Bick, A. G., Calvo, S. E. & Mootha, V. K. Evolutionary diversity of the mitochondrial calcium uniporter. *Science* **336**, 886, doi:10.1126/science.1214977 (2012).

11 Altschul, S. F., Gish, W., Miller, W., Myers, E. W. & Lipman, D. J. Basic local alignment search tool. *J Mol Biol* **215**, 403-410, doi:10.1016/S0022-2836(05)80360-2 (1990).

12 Ashburner, M. *et al.* Gene ontology: tool for the unification of biology. The Gene Ontology Consortium. *Nat Genet* **25**, 25-29, doi:10.1038/75556 (2000).

13 Fuchs, E. Keratins and the skin. *Annual review of cell and developmental biology* **11**, 123-153, doi:10.1146/annurev.cb.11.110195.001011 (1995).

14 Lopez-Pajares, V. *et al.* A LncRNA-MAF:MAFB transcription factor network regulates epidermal differentiation. *Dev Cell* **32**, 693-706, doi:10.1016/j.devcel.2015.01.028 (2015).
