# Supplementary material for: A data mining paradigm for identifying key factors in biological processes using gene expression data
Source: Sci Rep. 2018 Jun 13;8:9083. doi: 10.1038/s41598-018-27258-8 (PMC5998123; doi:10.1038/s41598-018-27258-8)
Supplement: Supplementary file 5 — Table S7 [file 41598_2018_27258_MOESM5_ESM.doc]

**Table S7. Primers and probes in RT-PCR analysis of atopic dermatitis.**

Universal ProbeLibrary for Human **Roche**

[**NM_001166034.1**](https://qpcr.probefinder.com/showsequence.jsp?seqNo=1541751051) **Homo sapiens suprabasin (SBSN), transcript variant 1, mRNA**
[**NM_198538.3**](https://qpcr.probefinder.com/showsequence.jsp?seqNo=672229455) **Homo sapiens suprabasin (SBSN), transcript variant 2, mRNA**
[**NM_001166035.1**](https://qpcr.probefinder.com/showsequence.jsp?seqNo=248983318) **Homo sapiens suprabasin (SBSN), transcript variant 3, mRNA**

##### Assay details:

| Use Universal ProbeLibrary probes: **#38, #38, #38** | | | | | | | |
| --- | --- | --- | --- | --- | --- | --- | --- |
| **Input** | **Probe ID** | **Primer** | **Length** | **Position** | **Tm** | **%GC** | **Sequence** |
| NM_001166034.1 | **#38 cat.no. 04687965001** | Left | 20 | 1450 - 1469 | 59 | 55 | gggagttcaccatacccttg |
| Multiplex with G6PD [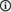](https://qpcr.probefinder.com/hkginfo.jsp?hkginfo=true&hkgNo=1252103166) |  | Right | 18 | 1493 - 1510 | 60 | 56 | gaacccttggaccgcttt |
|  |  | Amplicon (61 nt)  gggagttcaccatacccttgaacaggccgggaaggaagcagacaaagcggtccaagggtt  c | | | | | |
| NM_198538.3 | **#38 cat.no. 04687965001** | Left | 18 | 426 - 443 | 59 | 56 | tcaacaacgctgctggac |
| Multiplex with G6PD [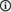](https://qpcr.probefinder.com/hkginfo.jsp?hkginfo=true&hkgNo=1252103166) |  | Right | 18 | 473 - 490 | 59 | 61 | cccagtgtggaacccttg |
|  |  | Amplicon (65 nt)  tcaacaacgctgctggacaggccgggaaggaagcagacaaagcggtccaagggttccaca  ctggg | | | | | |
| NM_001166035.1 | **#38 cat.no. 04687965001** | Left | 21 | 375 - 395 | 59 | 48 | accatggtattggacaagcag |
| Multiplex with G6PD [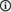](https://qpcr.probefinder.com/hkginfo.jsp?hkginfo=true&hkgNo=1252103166) |  | Right | 20 | 449 - 468 | 59 | 45 | gaagatccgctttgatggtt |
|  |  | Amplicon (94 nt)  accatggtattggacaagcaggaaaggaagcagagaagcttggccatggggtcaacaacg  ctgctggacagggcaaccatcaaagcggatcttc | | | | | |
